# The therapeutic potential of targeting chemokine signalling in the treatment of chronic pain

**DOI:** 10.1111/jnc.13927

**Published:** 2017-02-24

**Authors:** Karli Montague, Marzia Malcangio

**Affiliations:** ^1^ Wolfson Centre for Age‐Related DiseasesKing's College LondonLondonUK

**Keywords:** chemokines, chronic pain, proteases, therapy

## Abstract

Chronic pain is a distressing condition, which is experienced even when the painful stimulus, whether surgery or disease related, has subsided. Current treatments for chronic pain show limited efficacy and come with a host of undesirable side‐effects, and thus there is a need for new, more effective therapies to be developed. The mechanisms underlying chronic pain are not fully understood at present, although pre‐clinical models have facilitated the progress of this understanding considerably in the last decade. The mechanisms underlying chronic pain were initially thought to be neurocentric. However, we now appreciate that non‐neuronal cells play a significant role in nociceptive signalling through their communication with neurons. One of the major signalling pathways, which mediates neuron/non‐neuronal communication, is chemokine signalling. In this review, we discuss selected chemokines that have been reported to play a pivotal role in the mechanisms underlying chronic pain in a variety of pre‐clinical models. Approaches that target each of the chemokines discussed in this review come with their advantages and disadvantages; however, the inhibition of chemokine actions is emerging as an innovative therapeutic strategy, which is now reaching the clinic, with the chemokine Fractalkine and its CX_3_ CR_1_ receptor leading the way.

This article is part of the special article series “Pain”.

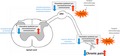

**Abbreviations used**
CCL: chemokine (C–C motif) ligand
CIPN: chemotherapy‐induced painful neuropathy
DRG: dorsal root ganglion
IL: interleukin
MCP‐1: monocyte chemoattractant protein 1
PSNL: partial sciatic nerve ligation

Chronic pain is a distressing, debilitating condition, which is poorly managed by clinically available drugs at present. It manifests in a variety of ways, with patients experiencing spontaneous pain, pain in response to innocuous stimuli (allodynia), heightened sensitivity to noxious stimuli (hyperalgesia) or irregular, unpleasant sensations (dysesthesia). Chronic pain can accompany a variety of injuries and conditions that result in lesions or dysfunction of the somatosensory nervous system and can continue for months or years after the initial tissue damage has healed. Such injuries include peripheral nerve injury (post‐surgical); damage to the central nervous system as a result of conditions such as multiple sclerosis (Watson and Sandroni 2016); injury resulting from viral infections (Uebelacker *et al*. 2015); metabolic disorders, such as diabetes (Davies *et al*. 2006); autoimmune disorders, such as rheumatoid arthritis (Wolfe *et al*. 2011) and injury as a result of drug treatment, such as chemotherapy (Dougherty *et al*. 2007).

Current treatments for chronic pain come with a host of undesirable side‐effects and provide only limited relief to patients. Because of the fact that the underlying mechanisms are not fully understood, effective therapies have yet to be developed. Substantial advances in our understanding of the mechanisms underlying chronic pain have been facilitated by murine pre‐clinical studies, which model chronic pain through surgical, pharmacological and immunization methods.

Chronic pain was historically attributed to a purely neuronal response to injury, which resulted in the development of ‘neurocentric’ strategies, in other words, therapies that focused on targeting neurons. However, extensive pre‐clinical evidence has now implicated significant contributions of non‐neuronal cells in the regulation of chronic pain and, in particular, has identified a pivotal role of neuron/non‐neuronal cell cross‐talk in the modulation of nociceptive signalling. The development of alternative therapeutic strategies has therefore now shifted focus towards targeting the activation of non‐neuronal cells, as well as the multitude of factors released by them, in response to nerve and tissue injury. Although the ultimate aim of developing new therapies is to dampen the neuronal signalling associated with pain, the novelty now comes with the target, which is no longer neurocentric.

One of the major ligand/receptor partnerships that facilitate communication between neurons and neighbouring non‐neuronal cells in the nervous system is chemokine signalling (Ramesh *et al*. 2013). Chemokines, or ‘chemotactic cytokines’, which were first identified over two decades ago as mediators of leucocyte migration (Oppenheim *et al*. 1991), are a family of small proteins, typically ranging from 8 to 17 kDa in size. Chemokines are classified according to the organization of cysteine residues on their N‐terminal region and are thus divided into four subfamilies: C, CC, CXC and CX_3_C (Luster 1998; Bajetto *et al*. 2002; Laing and Secombes 2004). Chemokines can be released by a variety of cell types in the central nervous system such as microglia, astrocytes and neurons (Bajetto *et al*. 2002). Various cell types in the peripheral nervous system also have the capacity to express chemokines, for instance, nociceptive sensory neurons in the dorsal root ganglia (Miller *et al*. 2008) as well as infiltrating monocytes/macrophages and Schwann cells, which constitute the myelin sheath surrounding axons (Kopydlowski *et al*. 1999; Saika *et al*. 2012).

While expressed in multiple cell types, in some cases the expression of a specific chemokine is restricted to a particular cell type, for instance, CX_3_CL_1_ is principally expressed by neurons (Clark *et al*. 2009). In most cases, signalling between chemokines and their receptors is promiscuous with one chemokine having the capacity to activate multiple chemokine receptors. For example, chemokine (C–C motif) ligand (CCL)3 is able to activate chemokine receptor (CCR)1, 5 and 9 (Kunkel 1999). In addition, one chemokine receptor can be activated by multiple chemokines. For instance, the CCR_5_, is activated by multiple chemokines (Combadiere *et al*. 1996; Raport *et al*. 1996; Samson *et al*. 1996; Nibbs *et al*. 1999).

The majority of chemokines are not constitutively expressed in the nervous system but are instead induced during adverse conditions (Bajetto *et al*. 2002). The latter feature makes chemokines particularly useful as therapeutic targets, because of the fact that their inhibition would be less likely to interfere with homeostatic processes, thus reducing adverse side‐effects. In addition, chemokine receptors are G protein‐coupled receptors, which are the most commonly targeted receptor class in modern medicine (Wells *et al*. 2006).

Chemokines have recently been associated with a variety chronic pain conditions clinically. For instance, the homeostatic chemokine CX_3_CL_1_, has been reported to be elevated in the bone marrow of rheumatoid arthritis patients (Kuca‐Warnawin *et al*. 2016). In addition, chemokines such as CCL_2_ and CCL_17_ have been observed in blood samples taken from temporomandibular disorder and fibromyalgia patients respectively (Slade *et al*. 2011; García *et al*. 2013). This highlights the importance and validity of investigating the role in chemokines in chronic pain disorders. Here, we discuss pre‐clinical evidence for the role of a selected number of chemokines in neuron‐non‐neuronal cell communication in the context of chronic pain and consider the therapeutic potential of disrupting their signalling.

## CX_3_CL_1_/fractalkine

### Expression and distribution

**Figure 1:**
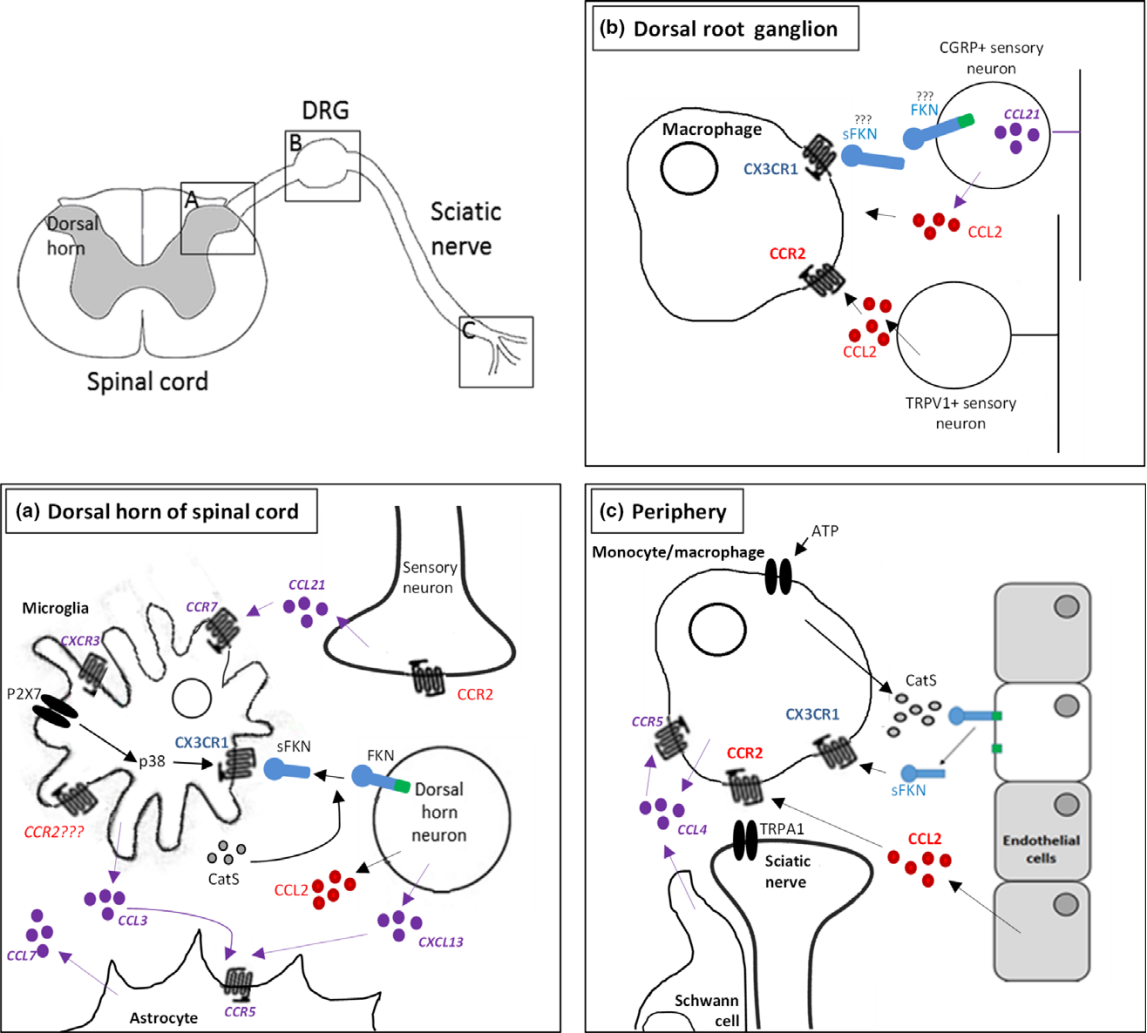
Schematic representation of chemokine/chemokine receptor expression in the dorsal horn of the spinal cord and the peripheral nervous system. Chemokines shown in purple have been less extensively studied. (a) The first pain synapse in the dorsal horn. A major signalling partnership that regulates neuron‐microglia communication in nociception is fractalkine (FKN)/CX_3_ CR_1_ signalling. FKN is expressed exclusively on neurons in the dorsal horn and is cleaved by Cathepsin S (CatS), which is released by neighbouring microglia, to produce soluble FKN (sFKN). sFKN activates CX_3_ CR_1_ receptors expressed by microglia. Dorsal horn neurons have also been shown to express chemokine (C–C motif) ligand 2 (CCL_2_), however, the expression of the chemokine receptor (CCR)2 receptor, is unclear. Dorsal horn neurons also express CXCL_13_, which activates CCR_5_ receptors expressed by astrocytes. CCR_5_ receptors are also activated by CCL_3_, which is expressed by microglia. Afferent nerve terminals in the dorsal horn also express chemokines associated with chronic pain, specifically CCL_21_, which has the capacity to activate both CXCR3 and CCR7 receptors expressed by microglia, the latter of which is induced in chronic pain. (b) In the dorsal root ganglion, FKN has been reported to be expressed by sensory neurons (however, this is controversial, see Kim *et al*. 2011). sFKN activates CX_3_ CR_1_ receptors, which are expressed by macrophages. Macrophages in the dorsal root ganglion (DRG) also express the CCR_2_ receptor, which is activated by CCL_2_ released from both CGRP and TRPV_1_‐positive sensory neurons. CGRP‐positive sensory neurons also express CCL_21_, although CCL_21_ actions in the DRG are not established. (c) In the periphery, FKN is expressed by endothelial cells, and is cleaved by CatS, which is found in monocytes/macrophages. sFKN activates CX_3_ CR_1_ receptors, also expressed by monocytes/macrophages. Both macrophages and Schwann cells also express CCL_4_, which can activate CCR_5_ receptors expressed by macrophages. Endothelial cells also express CCL_2_, which activates CCR_2_ receptors in monocytes/macrophages.

CX_3_CL_1_, otherwise known as fractalkine (FKN), is the only member of the CX_3_C subfamily of chemokines. In the CNS, FKN is expressed predominantly by neurons and unlike the majority of chemokines, is expressed constitutively. Indeed, in the spinal cord, expression of FKN is restricted only to neurons under basal conditions (Lindia *et al*. 2005; Clark *et al*. 2009; Yang *et al*. 2012). In the peripheral nervous system, FKN expression has been observed in the cell bodies of sensory neurons in the dorsal root ganglia (Verge *et al*. 2004). The expression of FKN at the central terminals of these neurons in the spinal cord, however, is debated with studies reporting both a presence (Verge *et al*. 2004) and absence (Clark *et al*. 2009) of FKN at this location. When the FKN expression profile was examined in a FKN reporter mouse (Kim *et al*. 2011), however, it was confirmed that neurons in the CNS did indeed express FKN, but sensory neurons in the dorsal root ganglion (DRG) as well as their terminals in the spinal cord, did not (Fig. 1). In the periphery, FKN is constitutively expressed in endothelial cells in the skin and intestine (Papadopoulos *et al*. 1999; Muehlhoefer *et al*. 2000) as well as in the heart (Harrison *et al*. 1999) and lungs (Foussat *et al*. 2000).

FKN exists as both a full length, membrane‐bound form of approximately 100 kDa in size, and a cleaved, soluble form (sFKN) of 80 kDa in size, making this chemokine an anomaly compared to other chemokines in terms of size, with other chemokines typically not exceeding 17 kDa. The cleavage of FKN is both constitutive and inducible. The membrane‐bound form serves an adhesion function in the context of the vascular immune system, and regulates the firm adhesion of leucocytes without the activation of integrins (Fong *et al*. 1998). sFKN, however, functions as a chemoattractant for T cells, B cells, Natural Killer (NK) cells and monocytes (Imai *et al*. 1997; Corcione *et al*. 2009) and is essential for the trans‐endothelial migration of monocytes that express the FKN receptor (Auffray *et al*. 2007). The cleavage of FKN and thus its shedding from the cell membrane is dependent on two classes of proteases, namely, metalloproteases (ADAM 10 and ADAM 17), which are expressed by endothelial cells (Hundhausen *et al*. 2007; Hurst *et al*. 2009) and the cysteine protease cathepsin S (CatS), which is released by microglia in the spinal cord (Clark *et al*. 2009). While ADAM 10 regulates the constitutive cleavage of FKN (Hundhausen *et al*. 2003), cleavage by ADAM 17 and CatS are induced in adverse conditions (Clark *et al*. 2009; Hurst *et al*. 2009). The ADAMs and CatS target membrane‐bound FKN at different cleavage sites and therefore give rise to different forms of sFKN, which could serve subtly different functions.

Unlike other, typically promiscuous, chemokines, FKN exclusively activates the CX_3_CR_1_ receptor. CX_3_CR_1_ was initially identified in humans and rats around 20 years ago (Harrison *et al*. 1994; Imai *et al*. 1997) and is a seven transmembrane domain G protein‐coupled receptor, specifically of the Gi and Gz subtypes (Al‐Aoukaty *et al*. 1998). The expression profile of CX_3_CR_1_ has been most extensively characterized using the CX_3_CR_1_‐GFP reporter mouse line, with the receptor present in murine monocytes (CD11b^+^Gr1^low^), NK cells, myeloid and lymphoid dendritic cells and in a subset of cutaneous Langerhans cells (Jung *et al*. 2000). In the nervous system specifically, CX_3_CR_1_ appears to be exclusively expressed in microglia in the spinal cord and brain (Lindia *et al*. 2005; Yang *et al*. 2012; Clark *et al*. 2013).

One caveat to our current understanding of FKN/CX_3_CR_1_ expression is that initial studies in which expression has been characterized have relied entirely on antibodies. In some cases, such antibodies have since been shown to be unreliable in the light of the development of transgenic mice, often giving false‐positives. It is therefore prudent to assume that expression patterns revealed by antibodies are only *bona fide* expression if confirmed in transgenic mice. Inconsistent findings regarding expression must be interpreted with caution and cannot form the basis of therapy development. Here, we will consider selected, critical studies that have used both antibodies and transgenic lines, in which both approaches have been in agreement with each other. The distribution of FKN and CX_3_CR_1_ in the spinal cord, which has indeed been confirmed by both antibodies and the use of transgenic mice, is predominantly neuronal and microglial respectively. This strongly indicates that this signalling pair has the potential to mediate neuron‐microglial communication, both homeostatic and pathological.

## CX_3_CL_1_/R1 signalling in pre‐clinical models of chronic pain

### Central mechanisms

The first synapse in the nociceptive pathway – between the terminals of the primary afferents and dorsal horn neurons in the spinal cord – is a key site at which the modulation of nociceptive signalling occurs. It is well established that damage to peripheral nerves, such as the sciatic nerve, disrupts homoeostasis and consequentially results in heightened response states of microglia and astrocytes in the spinal cord (McMahon and Malcangio 2009). This heightened microglial activity results in an increase in neuron‐microglial communication, which has the capacity to amplify nociceptive transmission, resulting in a chronic pain state. In the dorsal horn of the spinal cord, the distribution of FKN and CX_3_CR_1_, neuronal and microglial, respectively, makes this signalling pair an ideal candidate for the mediation of this increase in communication. Indeed, in the last decade, pre‐clinical studies have advanced our understanding of, and established the role of FKN/CX_3_CR_1_ signalling in chronic pain; thus, bringing the therapeutic potential of targeting this signalling pathway into prominence.

FKN, sFKN in particular, has been shown to be pronociceptive, with intrathecal administration of the FKN domain, but not the full length FKN, resulting in both thermal and mechanical hypersensitivity (Clark and Malcangio 2012; Clark *et al*. 2007; Zhuang *et al*. 2007). This effect is mediated by CX_3_CR_1_ activation (Clark *et al*. 2007; Staniland *et al*. 2010), which in turn induces intracellular phosphorylation of microglial p38 MAPK (Clark *et al*. 2007; Zhuang *et al*. 2007) resulting in the release of proinflammatory mediators such as Interleukin 1β (IL‐1β), Interleukin 6 (IL‐6) and nitric oxide (Milligan *et al*. 2005).

Because of the pronociceptive nature of sFKN/CX_3_CR_1_ signalling, the inhibition of this pathway is intuitively a potential therapeutic strategy for the treatment of chronic pain. Indeed, changes in FKN/CX_3_CR_1_ signalling in pre‐clinical models of chronic pain as well as the therapeutic potential of disrupting such signalling, has been extensively investigated. Following injury to peripheral nerves, the expression of CX_3_CR_1_ in spinal microglia increases significantly (Zhuang *et al*. 2007; Staniland *et al*. 2010). It is important to keep in mind, however, that the elevated levels reported could be because of proliferation of CX_3_CR_1_‐expressing microglia and therefore increased expression of CX_3_CR_1_‐expressing cells as opposed to elevated CX_3_CR_1_ expression *per se*. Although the general consensus is that the expression of total (membrane‐bound and cleaved) FKN remains unchanged following peripheral nerve injury (Clark *et al*. 2009; Old *et al*. 2014), levels of sFKN specifically in CSF do appear to increase (Clark *et al*. 2009; Nieto *et al*. 2016). Chronic pain models are therefore accompanied by an increase in both sFKN specifically and CX_3_CR_1_.

In various pre‐clinical models of peripheral nerve injury, the intrathecal administration of neutralizing antibodies against either FKN or CX_3_CR_1_ have been found to delay or attenuate chronic pain‐associated behaviours (Milligan *et al*. 2004; Clark *et al*. 2007; Zhuang *et al*. 2007) through a reduction in p38 MAPK phosphorylation in microglia and, in turn, a reduction in the release of proinflammatory cytokines and dampening of neuron–glia communication (Zhuang *et al*. 2007). The therapeutic benefits of CX_3_CR_1_ inhibition and thus reduced phosphorylation of microglial p38 MAPK, also extend to other models of chronic pain, such as bone cancer, which is also accompanied by microgliosis and elevation of microglial CX_3_CR_1_ and p38 MAPK (Yin *et al*. 2010; Hu *et al*. 2012). Indeed, intrathecal delivery of a CX_3_CR_1_ neutralizing antibody delayed chronic pain‐associated behaviour in this model as well as reduced microgliosis and the associated increases in CX_3_CR_1_ and p38 phosphorylation.

The role of FKN/CX_3_CR_1_ modulation of neuron‐microglial signalling in chronic pain has also been investigated using CX_3_CR_1_ knock‐out mice, which consistently display deficits in chronic pain in various pre‐clinical models, whether they are traumatic or non‐traumatic. For example, CX_3_CR_1_ knock‐out mice that are subjected to partial sciatic nerve ligation (PSNL) display reduced mechanical and thermal hypersensitivity compared to wild‐type mice that undergo the same procedure (Staniland *et al*. 2010). This deficit in chronic pain‐associated behaviour observed in CX_3_CR_1_ knock‐out mice is correlated with a reduction in microglial activity. In the case of PSNL, the infiltration of macrophages in the sciatic nerve, the site of injury, was not altered in CX_3_CR_1_ knock‐out mice (Staniland *et al*. 2010), and so it appears that the reduction in pain behaviour observed in these animals is mediated specifically by CX_3_CR_1_‐dependent microglial activity in the spinal cord.

### The role of CX_3_CL_1_/FKN cleavage by CatS in chronic pain

Because of the fact that sFKN specifically has been found to increase following peripheral nerve injury and is pronociceptive, changes in CatS, which regulates cleavage and hence shedding of FKN to form sFKN, has also received attention in recent years in the context of chronic pain pre‐clinical models. Following injury to peripheral nerves, CatS expression increases in microglia that are located in areas of the spinal cord that are innervated by the injured peripheral afferents (Clark *et al*. 2007) and it is subsequently released from microglia downstream of P_2_X_7_ receptor signalling (Clark *et al*. 2010). This P_2_X_7_‐dependent release of CatS results in the shedding of sFKN from neurons in the dorsal horn, which in turn, signals to CX_3_CR_1_‐expressing microglia (Clark *et al*. 2007).

The pronociceptive effects of CatS have also been established *ex vivo*. For example, in spinal cord slices, which have been prepared from neuropathic animals and still have the damaged dorsal root attached, electrical stimulation of the injured dorsal roots results in the liberation of FKN, indicative of activated or elevated CatS (Clark *et al*. 2009). This liberation of sFKN appears to only be apparent under conditions in which the microglia are reactive, for example, injury or lipopolysaccharide (a macrophage activator) exposure (Clark *et al*. 2009). In addition, the pronociceptive effects of intrathecal delivery of CatS *in vivo* are prevented by neutralization of spinal FKN and in CX_3_CR_1_ knock‐out mice (Clark *et al*. 2007).

The therapeutic potential of inhibiting CatS appears to be most promising for the maintenance phase of chronic pain as opposed to the initial induction. Both systemic (Irie *et al*. 2008; Zhang *et al*. 2014) and intrathecal (Clark *et al*. 2007) delivery of a CatS inhibitor reversed established injury‐induced neuropathic pain, but was not effective if administered during the initial phase of pain (e.g. day 3 post‐surgery), at which time, CatS expression was relatively low in the spinal cord (Clark *et al*. 2007; Zhang *et al*. 2014) and peripherally (Barclay *et al*. 2007). Furthermore, CatS knock‐out mice demonstrate similar levels of chronic pain to wild‐type controls during initial phases of peripheral injury models but display lower levels of pain relative to wild‐type controls from day 3, at which point, pain is considered to have entered the maintenance phase (Zhang *et al*. 2014).

The inhibition of CatS has also been shown to have therapeutic potential in non‐surgical pre‐clinical models of chronic pain, for example, murine Rheumatoid Arthritis (RA). Rats with collagen‐induced arthritis displayed mechanical hypersensitivity as well as microglial activation and an elevation of IL‐1β in the CSF, all of which was prevented by intrathecal administration of N‐morpholinurea‐homophenylalanyl‐leucyl‐vinylsulfonemethyl (LHVS), a selective CatS inhibitor (Nieto *et al*. 2016). CatS‐mediated release of sFKN therefore appears to mediate chronic pain maintenance and could provide a therapeutic target that has potential where other current therapies have shown limited efficacy, acting as an alternative or complementary addition to currently available drugs. Indeed, in a recent study, the use of a CatS inhibitor has been shown to enhance the effects of gabapentin and pregabalin in a PSNL model in rats, as well as resulting in a dose‐dependent attenuation of injury‐induced allodynia when used alone (Hewitt *et al*. 2016).

### Peripheral mechanisms

FKN/CX_3_CR_1_ signalling in the spinal cord is a promising therapeutic target; however, it is now thought to not be the only location at which this signalling partnership can mediate chronic pain, with FKN/CX_3_CR_1_ signalling in the periphery also being implicated in models of chemotherapy‐induced painful neuropathy (CIPN). In paclitaxel‐treated rats for instance, the expression of FKN has been reported to increase in primary sensory neurons in the DRG *in vivo* and *in vitro* and macrophage infiltration into the DRG is reported to increase alongside the development of allodynia (Huang *et al*. 2014). Intrathecal administration of a FKN‐neutralizing antibody was shown to prevent macrophage recruitment to the DRG and reduced activation of p38 MAPK in macrophages in addition to attenuating paclitaxel‐induced allodynia (Huang *et al*. 2014). The role of FKN/CX_3_CR_1_ signalling in sensory neurons in the DRG, however, should be interpreted with caution because of the fact that FKN expression in the DRG is controversial, with contradictory expression patterns being reported, as discussed above. Indeed, the lack of evidence for sensory neuron expression in the FKN reporter transgenic line (Kim *et al*. 2011) suggests that FKN is unlikely to be expressed in neurons in the DRG and any analgesics effects of its inhibition could be because of CX_3_CR_1_ inhibition in macrophages in the DRG, (Old *et al*. 2014).

In an alternative model of CIPN, Vincristine‐induced neuropathic pain, FKN/CX_3_CR_1_ signalling in macrophages that have been recruited to the sciatic nerve following vincristine treatment has provided an additional therapeutic target (Old *et al*. 2014). Vincristine treatment triggers the recruitment of CX_3_CR_1_‐expressing monocytes/macrophages to the sciatic nerve, with CX_3_CR_1_ in this location being activated by endothelial FKN (Fig. 1). This results in the downstream activation of Transient receptor potential cation channel, subfamily A, member 1 (TRPA1) receptors in sensory neurons as well as the generation of reactive oxygen species (Old *et al*. 2014). In this model of CIPN, CX_3_CR_1_ knock‐out mice display a delayed onset in mechanical allodynia, which resembles the delay accompanying depletion of macrophages using low‐dose clodronate liposomes, and a decrease in infiltrating macrophages. Critically, no neuronal damage is observed in this model and there are no changes to microglial activity in the dorsal horn of the spinal cord, indicating that in this particular model, FKN/CX_3_CR_1_ signalling in the periphery as opposed to the spinal cord, plays a role in chronic pain (Old *et al*. 2014).

FKN/CX_3_CR_1_ signalling therefore provides a promising prophylactic target for the development of pain. Indeed the high fidelity of the partnership ensures that therapies targeted towards this signalling pair are highly specific relative to other therapeutic approaches. One caveat, however, is that unlike the majority of chemokines, FKN is constitutively expressed in neurons as well as a multitude of cells of the immune system suggesting that it also plays an essential role in homeostatic processes, which could be disrupted if signalling is inhibited and could therefore lead to side‐effects. This highlights the importance of targeting sFKN specifically, which could be achieved by targeting CatS, as opposed to full length FKN or CX_3_CR_1_. Nonetheless, the FKN/CX_3_CR_1_ signalling partnership provides unique therapeutic potential, which is already being appreciated in chronic pain conditions and indeed in other pathological conditions beyond pain, such as pancreatic disease (D'Haese *et al*. 2010).

## CCL_2_/MCP‐1

### Expression and distribution

Another chemokine, which has received much attention in the context of chronic pain is CCL_2_. CCL_2_ or monocyte chemoattractant protein 1 (MCP‐1) recruits monocytes to the site of infection, ischaemia or inflammation. While CCL_2_ is able to recognize the receptors CCR1, 2 and 4, it shows preference for CCR_2_ (Jung *et al*. 2009). The expression of CCL_2_ in primary sensory neurons has been well studied. Under basal conditions, low levels of CCL_2_ are constitutively expressed in more than 40% of small and medium DRG neurons, with co‐expression being observed with substance P, Calcitonin gene‐related peptide (CGRP) and the Transient receptor potential cation channel, subfamily V, member 1 (TRPV_1_) (Tanaka *et al*. 2004; Dansereau *et al*. 2008). The expression of CCL_2_ has been found to increase in several pathological conditions. For instance, following PSNL, the expression of CCL_2_ increased rapidly in DRG neurons, within 4 h of the injury occurring (Tanaka *et al*. 2004). Likewise, following nerve constriction, CCL_2_ expression was found to be induced in both small and large diameter neurons in the DRG that also expressed ATF3, an indicator of neuronal injury (Zhang and De Koninck 2006). The expression of the CCR_2_ receptor, for which CCL_2_ has the highest affinity, has also been well documented in several models of chronic pain. For example, *in situ* hybridization shows that chronic constriction of the DRG results in an increase in the expression of CCR_2_ mRNA in both neuronal and satellite cells in the compressed DRG as well as the adjacent, non‐compressed DRG (White *et al*. 2005). An increase in CCR_2_ expression has also been observed in the DRG following sciatic nerve demyelination (Jung *et al*. 2009).

Expression of CCL_2_/R_2_ in the spinal cord, however, is controversial (Fig. 1). CCL_2_ has been reported to be constitutively expressed in a variety of cell types, having been observed in primary afferent neurons (Zhang and De Koninck 2006) as well as co‐localizing with the astrocytic marker, Glial fibrillary acidic protein (GFAP) (Gao *et al*. 2009). Expression in astrocytes appears to increase following the induction of different injury models, for example, spinal nerve ligation (Gao *et al*. 2009), spinal cord contusion injury (Knerlich‐Lukoschus *et al*. 2008) and demyelinating lesions (Van Der Voorn *et al*. 1999). Thus, CCL_2_ expression in astrocytes appears to be elevated in the spinal cord following neuronal injury, regardless of the nature. The expression profile of CCR_2_ remains debated, with different detection methods producing conflicting results. In CCR_2_‐GFP reporter mice, for example, a weak but positive GFP signal is apparent in dorsal horn neurons. *In situ* hybridization, however, suggests an absence of CCR_2_ mRNA in the spinal cord under basal conditions (Jung and Miller 2008). What has been more consistently reported, however, is the elevation of CCR_2_ in the spinal cord following nerve injury. For instance, an increase in CCR_2_ mRNA in the deep dorsal horn and motor neurons has been reported 3 days after spinal nerve injury (Gao *et al*. 2009). Furthermore, an elevation in CCR_2_ has been reported in spinal microglia following sciatic nerve injury (Abbadie *et al*. 2003; Thacker *et al*. 2009) as well as in astrocytes following spinal cord contusion (Knerlich‐Lukoschus *et al*. 2008). However, the reliability of antibodies used to detect the presence of CCR_2_ has been recently doubted and the precise expression profile of CCR_2_ in the spinal cord has yet to be firmly established.

The fact that both CCL_2_ and CCR_2_ have previously been suggested to increase in pre‐clinical models of pain and were initially thought to be located in neurons and microglia, respectively (although expression is debatable and not exclusive to one cell type), makes this signalling pair another potential player in the mechanisms underlying chronic pain, which has received considerable attention over the last decade.

### CCL_2_/R_2_ signalling in pre‐clinical models of chronic pain

Initial studies investigating CCL_2_/CCR_2_ signalling in the spinal cord suggested that this signalling pair played a considerable role in neuron‐microglial communication in chronic pain animal models. For instance, in *ex vivo* preparations of the dorsal horn of the spinal cord, CCL_2_ levels obtained from spinal cord superfusates, while similar in unstimulated naive and neuropathic animals, were only elevated as a result of supramaximal electrical stimulation of the dorsal root in neuropathic animals, which also displayed heightened mechanical hypersensitivity (Thacker *et al*. 2009). In addition, application of intraspinal CCL_2_ in naïve rats resulted in chronic pain‐like behaviour, while intrathecal delivery of a CCL_2_‐neutralizing antibody inhibited injury‐associated pain behaviour (Thacker *et al*. 2009). This suggests that CCL_2_ is released in an activity‐dependent manner and in turn is able to regulate nociceptive signalling.

CCL_2_ has also been suggested to play a role in non‐traumatic pre‐clinical models of chronic pain, such as CIPN. In the paclitaxel model of CIPN, for instance, CCL_2_ is reported to be elevated in the spinal cord of paclitaxel‐treated mice, which display paclitaxel‐induced cold hyperalgesia. Intrathecal administration of an antibody against CCL_2_ not only completely suppressed the hyperalgesia, but also inhibited paclitaxel‐induced microglial activation (Pevida *et al*. 2013). This suggests that cold hyperalgesia associated with paclitaxel therapy could be treated by targeting CCL_2_‐mediated activation of microglia (either directly or indirectly). CCL_2_ signals through both CCR_2_ and CCR1 and while CCR_2_ inhibition appears to alleviate paclitaxel‐induced allodynia, CCR1 inhibition does not (Pevida *et al*. 2013). This suggests that the pronociceptive effects of CCL_2_ are mediated via the CCR_2_ receptor. A caveat to this finding, however, is that the CCR_2_ inhibitor in the paclitaxel mouse model has been shown to have additional effects on NMDA receptor subunit expression and neuronal nitric oxide synthase (Ren *et al*. 2015). Both neuronal nitric oxide synthase and the NMDA receptor subunit; NR2B have been found to increase and be associated with pain‐like behaviour in a model of bone cancer chronic pain and they are down‐regulated in parallel to the alleviation of allodynia when the CCR_2_ inhibitor is administered (Ren *et al*. 2015). It is therefore possible that the analgesic effects of CCR_2_ inhibitors in some pre‐clinical models are not because of the inhibition of CCL_2_/R_2_ signalling, but additional, non‐specific effects, which could weaken the prominence of CCL_2_ signalling in the spinal cord in the underlying mechanisms of pain.

CCL_2_ expressed elsewhere, however, for example, the DRG, could still be involved in mechanisms underlying other forms of pain that are induced by paclitaxel. Paclitaxel has been shown to induce the expression of CCL_2_ and CCR_2_ in the primary sensory neurons of the DRG, specifically small, nociceptive neurons and medium‐sized neurons respectively (Zhang *et al*. 2013). Local blockade of CCL_2_/R_2_ signalling using either a neutralizing antibody against CCL_2_ or a CCR_2_ antisense oligodeoxynucleotide significantly reduced paclitaxel‐ target for CIPN.

While there is strong associated allodynia (Zhang *et al*. 2013), suggesting that neuronal CCL_2_/R_2_ signalling in the DRG could provide a therapeutic evidence to suggest that CCL_2_/R_2_ signalling in the DRG, and potentially the spinal cord, could play a role in chronic pain, the debatable and non‐exclusive expression profiles of both CCL_2_ and CCR_2_ currently limits their potential as therapeutic targets and this signalling partnership is in need of further investigation and clarification in order to be translated to the clinic.

### Other chemokines in neuropathic pain

Although CX_3_CL_1_/FKN and CCL_2_ are the most extensively studied chemokines in the context of chronic pain, other chemokines are now emerging as potential regulators and thus provide the opportunity for more novel therapies to be developed.

## CCL_7_/MCP‐3

CCL_7_, otherwise known as MCP‐3, has been shown in the last few years to play a role in the development of chronic pain in some pre‐clinical models. Following partial sciatic nerve ligation, the expression of CCL_7_ is elevated in astrocytes located in the dorsal horn of the spinal cord up to 2 weeks following the day of surgery (Imai *et al*. 2013). This elevation appears to be dependent on the proinflammatory cytokine; IL‐6, with IL‐6 knock‐out mice showing no changes in CCL_7_ expression after PSNL. Indeed, intrathecal delivery of recombinant IL‐6 into IL‐6 knock‐out mice results in an increase in CCL_7_ mRNA. Both *in vitro* and *in vivo* CCL_7_ treatment results in microglial activation, which is suppressed *in vivo* by intrathecal administration of a neutralizing antibody against CCL_7_. In addition, to the suppression of microglial activation, pain behaviours are also reduced (Imai *et al*. 2013). Because of the expression pattern of CCL_7_, it is therefore possible that it mediates an astrocyte‐microglia interaction in the context of chronic pain. Indeed, this has recently been shown to be the case in a spinal nerve ligation model of chronic pain (Ke *et al*. 2016). Following spinal nerve ligation, CCL_7_ expression in astrocytes in the dorsal horn of the spinal cord increased in correlation with the development of pain. Silencing CCL_7_ reduced surgery‐induced pain and *in vitro*, CCL_7_ has been shown to be sufficient for the proliferation of astroglial cells (Ke *et al*. 2016). *CCL*_7_ is therefore suggested to be essential for the development of chronic pain via the promotion of astrocyte proliferation.

## CCL_3_ and CCL_4_/MIP‐1b

CCL_3_ has also been recently suggested to play a role in the development of chronic pain in both traumatic (surgical) and non‐traumatic (non‐surgical) pre‐clinical models. In a model utilizing chronic constriction injury of the sciatic nerve, both CCL_3_ and its receptor CCR_5_ were found to increase alongside microglial activation and the development of both thermal and mechanical hypersensitivity (Sun *et al*. 2016). Intrathecal delivery of a CCL_3_‐neutralizing antibody suppressed the up‐regulation of proinflammatory cytokines such as Tumor necrosis factor alpha (TNFα) and IL‐1β as well as the activation of microglial p38 MAPK, indicating that CCL_3_ regulates, at least in part, the release of proinflammatory cytokines and microglial activation. Furthermore, in CCR_5_ knock‐out mice, pain behaviour was suppressed (Sun *et al*. 2016). It is important to keep in mind, however, that CCR_5_ is not exclusively activated by CCL_3_ and the effects of the CCR_5_ knockout could be a result of disrupting the signalling of other chemokines such as CCL_4_ and CXCL_13_.

CCL_3_ has also been implicated in non‐surgical models of chronic pain, for instance, CIPN. Following the treatment of rats with paclitaxel, and the consequential development of mechanical allodynia, microglial activation was reported to increase in the dorsal horn of the spinal cord along with mRNA for both CCL_3_ and CCR_5_ (Fig. 1; Ochi‐ishi *et al*. 2014). The increase in CCL_3_ is potentially a result of paclitaxel‐induced increase in P_2_X_7_ receptors, which are suggested to trigger the release of CCL_3_ from microglia (Kataoka *et al*. 2009). Intrathecal administration of a CCL_3_‐neutralizing antibody not only appeared to attenuate paclitaxel‐induced allodynia, but reverse allodynia during the maintenance phase, indicating that CCL_3_ could play a role in both induction and maintenance of chronic pain potentially making CCL_3_ a versatile therapeutic target.

CCL_4_, otherwise known as macrophage inflammatory protein 1b shares the CCR_5_ receptor with CCL_3_ and has also been implicated in the regulation of chronic pain in recent years. Following the induction of a partial sciatic nerve ligation, CCL_4_ mRNA increases in both macrophages and Schwann cells in the periphery as early as day 1 post‐surgery (Fig. 1; Saika *et al*. 2012). Following local administration of a CCL_4_‐neutralizing antibody to the region surrounding the sciatic nerve, both mechanical and thermal allodynia that occurred as a result of surgery, were prevented. In addition, PSNL‐induced up‐regulation of inflammatory chemo/cytokines was also prevented, suggesting that CCL_4_ could potentially play a peripheral role in the regulation of chronic pain *via* regulation of proinflammatory chemo/cytokine release. In addition, administration of the CCR_5_ antagonist also prevented allodynia in this model (Saika *et al*. 2012). As discussed, however, CCR_5_ is activated by multiple chemokines and therefore targeting CCR_5_ will lack the specificity that targeting CCL_3_ or CCL_4_, specifically would have.

## CCL_21_

CCL_21_, another member of the CC subfamily, is another chemokine that has been suggested to play a role in the mechanisms underlying chronic pain. Following peripheral nerve injury, CCL_21_ expression is rapidly induced in small‐diameter primary sensory neurons and is transported to central terminals in the dorsal horn, where it is suggested to increase the expression of the P_2_X_4_ receptor in microglia (Biber *et al*. 2011). Intrathecal administration of a CCL_21_‐blocking antibody reduced injury‐induced allodynia suggesting that it, at least in part, mediates nociceptive signalling in this pre‐clinical model. Indeed, CCL_21_ knock‐out mice do not develop allodynia and in these mice, P_2_X_4_ receptor expression in microglia does not significantly increase following injury compared to control mice. Critically, CCL_21_ injection into CCL_21_ knock‐out mice, results in the same development of injury‐induced allodynia as that seen in wild‐type controls (Biber *et al*. 2011). In addition, CCL_21_ administration *in vivo* as well as application to microglia *in vitro*, is sufficient to increase microglial P_2_X_4_ receptor expression. CCL_21_ has been found to activate both the CXCR3 and CCR7 receptors, which are expressed by microglia constitutively and following microglial activation respectively (Biber *et al*. 2001; Rappert *et al*. 2002; Dijkstra *et al*. 2004). While knocking out CXCR3 appears to have no effect on injury‐induced mechanical allodynia, CCR7 knock‐outs display a delayed onset of allodynia (Biber *et al*. 2011), suggesting that pronociceptive actions of CCL_21_ are more likely to be elicited via activation of the CCR7 receptor.

## CXCL_13_

The most recent chemokine to receive attention in the context of chronic pain is CXCL_13_. In the last year, CXCL_13_ has been shown to regulate chronic pain in both partial infraorbital nerve ligation, resulting in trigeminal neuropathic pain (Zhang *et al*. 2016) and spinal nerve ligation (Jiang *et al*. 2016). In the former, both CXCL_13_ and its receptor CCR_5_ were found to increase, with the inhibition of either using a shRNA lentivirus, attenuating allodynia. In the latter, CXCL_13_ expression was found to increase in the spinal cord, and again, suppression of expression using a shRNA lentivirus reduced the injury‐associated allodynia. The potential regulation of pain by CXCL_13_ has been tentatively suggested to occur via the activation of astrocytes in the dorsal horn, with intrathecal injection of CXCL_13_ resulting in the activation of astrocytes in naive mice (Jiang *et al*. 2016).

## Conclusion

Chronic pain is a debilitating condition, which is experienced even when the painful stimulus has been resolved. At present, clinically available treatments for chronic pain have limited efficacy and undesirable side‐effects. More effective, novel therapeutic approaches are therefore needed. Although the mechanisms underlying chronic pain were initially thought to be neurocentric, it is now accepted that non‐neuronal cells also play a role in nociceptive signalling and the communication between neurons and non‐neuronal cells in the context of chronic pain has received more attention in the last decade. One of the signalling pathways known to mediate the communication between neurons and neighbouring non‐neuronal cells is chemokine signalling. Here, we considered a selection of chemokines (Fig. 1), which, along with their receptors, have been reported to increase in a variety of traumatic and non‐traumatic pre‐clinical models of chronic pain. Their therapeutic potential has been realized in the light of the reversal of pain that accompanies their inhibition. Each chemokine that has been suggested to play a role in chronic pain comes with its promises and controversies in terms of the development of therapies. In the case of CX_3_CL_1_, for example, the high fidelity of its signalling and exclusive expression profile means that drugs that are targeted to this signalling partnership will have highly specific effects. While the debatable and variable expression profile of CCL_2_/R_2_ means that currently, targeted therapies will be difficult to develop.

At present, although chemokine signalling inhibitors are in their infancy in the clinic, interest has been rapidly expanding and chemokines, particularly CX_3_CL_1_ are becoming more recognized as innovative therapeutic targets for a number of pathological conditions. The CX_3_CL_1_ inhibitor, AZD8797, for example, which was originally developed for use in a pre‐clinical model of multiple sclerosis (Ridderstad Wollberg *et al*. 2014), has shown promise in terms of safety and toxicology in humans and its use in cancer pain may be being considered, signifying that a new chapter of drug development has already begun, with the CX_3_CL_1_/CX_3_CR_1_ signalling partnership leading the way.
